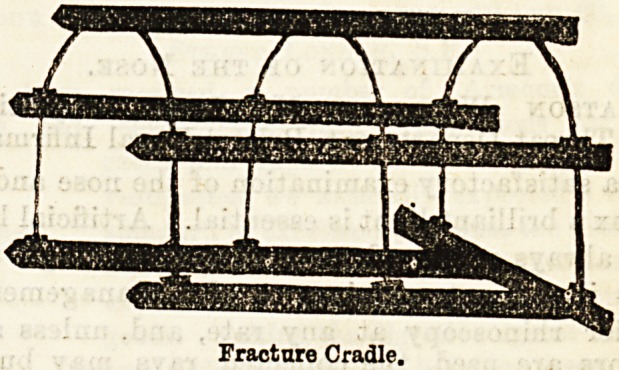# Treatment of His Simple Fracture of the Tibia and Fibula

**Published:** 1893-05-27

**Authors:** 


### THE HOSPITAL CLINIC.

*[The Editor will be glad to receive offers of co-operation and contributions from members of the profession. All letters should be addressed to* THE EDITOR, THE LODGE, PORCHESTER SQUARE, LONDON, W.]

### SUSSEX COUNTY HOSPITAL.

### TREATMENT OF SIMPLE FRACTURE OF THE TIBIA AND FIBULA.

The victims of this accident fall under the notice of the House Surgeon, after admission into the receiving room. The preliminary task of picking up the patient at the site of the accident, and conveying him to the hospital often devolves on persons more or less unacquainted with ambulance work.

Fortunately a considerable proportion of these cases are first seen and handled by the Brighton police, whose zeal and general knowledge of the principles of "first aid" cannot be too highly commended. In their hands the original injury is seldom made worse during transit to the hospital, and the ingenuity displayed by them in the matter of impromptu splints and bandaging is sometimes remarkable.

Constables' batons, broomsticks, pieces of packing-case or card-board, carpenters' rulers, the sound limb, and other strange articles too numerous to mention are brought into requisition with the laudable object of preventing, as far as possible, any further displacement of the broken fragments of the bones.

The House Surgeon, with the assistance of the students or nurse, exposes the damaged limb, either by gently drawing up the trowser leg or slitting up the outside seam with a pair of strong scissors. After satisfying himself as to the nature of the injury to the leg, and as to the existence of any further damage that the patient may have sustained, he reduces the displacement, and applies side splints with firm bandaging before removal into the Accident Ward. The fractures usually occur in the lower third of the leg, the fibula giving way an inch or more above the site of the tibial lesion.

The apparatus in every day use for the treatment of these fractures consists of the following items: 1. Hard horsehair mattress. 2. Tinned iron back splint with

Iron Splint.

foot piece, large oval hole for the heel, double curve for the calf and flexure of knee, and cross pieces for the suspensory straps; the whole thickly padded. 3. Well padded side splints about six inches in depth, and reaching the whole length of the back splint. 4. Two thick leather straps about six feet long. 5. Fracture cradle, consisting of wooden frame and iron bars. 6. Heel pads, a large assortment made of cotton wool covered with old linen. 7. Open wove bandages two and a-half inches broad.

These are placed at the bedside by the head nurse, whose duty it is to see that the splints are of the proper size, and that everything likely to be wanted is prepared, so that there may be no delay. The patient is then washed with the exception of the injured limb, which remains in the temporary side splints until the house surgeon's visit. If there is great pain or much starting of the limb, opium is administered either in the form of the tincture or as a hypodermic injection of morphine.

Unless there is much restlessness no anæsthetic is used while the limb is being put up. When the patient is washed and warmed with hot bottles the head nurse sends for the house surgeon. The assistant nurse now holds and steadies the foot on the injured side while the house surgeon removes the side splints and examines the fracture. The head nurse then carefully and gently washes the limb, first using tow saturated with turpentine, and afterwards hot soap and water. The next step consists in placing the limb on the back splint and adjusting the heel pads. Strong extension

Fracture Cradle.

is then made on the foot (1) to reduce displacement, (2) to bring the sole of the foot in contact with the foot-piece of the splint, (3) to fix the foot in a position at right angles to the leg. The foot is then held in position by the assistant nurse while the bandages are put on, the head nurse supporting the splint and leg, and helping to allay apprehension or restlessness on the part of the patient. Spasmodic contraction of the calf muscles at this stage and lack of co-operation in a nervous patient often makes this part of the operation difficult and tedious, especially in very muscular subjects. The foot bandage is put on as follows, having regard to the fact that the foot always tends to fall over towards the outer side: The loose end of the bandage is pinned firmly to the inner edge of the padding of the foot-piece of the splint; the bandage is then passed outwards between the sole and the foot-piece and round the foot from without inwards; the foot is then bandaged in the ordinary way with figure of 8 turns. By this method a sling is made, which tends to prevent the foot from becoming displaced. The knee is bandaged to the splint and as much of the leg as may be necessary to keep the fragments in good position. The correct adjustment of the heel pad or pads is relied upon mainly for bringing the fragments together and to prevent "riding" of the lower end of the upper fragment. The cradle is now placed over the limb and the straps are passed through slots in the splint and cradle, which are tightened so as to raise the splint clear of the bed, taking care that it lies horizontally. Lastly, the side splints are applied, and fastened with webbing straps. An opiate or draught of bromide and chloral is given for the first few nights. Lumbar pain is of frequent occurence during the first week, arising from the enforced dorsal decubitus; this is relieved to some extent by a soft pillow pushed under the small of the back. As a rule, it is found necessary to readjust the splints every two or three days until the patient has become accustomed to his position, and learns to keep the leg still.

Should the upper fragment remain prominent, in spite of readjustment of the heel pads and bandages, or raising the lower end of the splint by tightening the lower straps, recourse is sometimes had to india-rubber air pads measuring about four or five inches square in their collapsed condition. These can be readily inflated to any size or tension. They resemble in their collapsed state the large Barnes bags used in midwifery. One of these placed over the projecting fragment and fastened with a webbing strap often proves of great service. Bullae are usually left to themselves, unless they are very large, when they are pricked and treated with some antiseptic application. As soon as there are signs of bony union, the iron splint is removed and a plaster of paris splint substituted for it. The form of splint invented by Mr. Croft is now used in preference to others. as being more rapidly and easily applied, and readily admitting of removal and re-application. As soon as the patient can get about on crutches he is discharged, with instructions to return once a week for examination.

Plain wooden crutches made by the hospital carpenter are lent him for as long as they are needed.

## Figures and Tables

**Figure f1:**
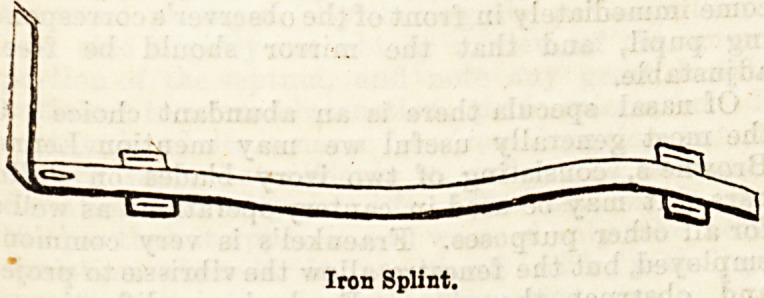


**Figure f2:**